# COVID-19 and cognitive impairment: neuroinvasive and blood‒brain barrier dysfunction

**DOI:** 10.1186/s12974-022-02579-8

**Published:** 2022-09-07

**Authors:** Yanting Chen, Wenren Yang, Feng Chen, Lili Cui

**Affiliations:** 1 Department of Neurology, Guangdong Key Laboratory of Age-Related Cardiac and Cerebral Diseases, Affiliated Hospital of Guangdong Medical University, Zhanjiang, 524000 China; 2 Department of Trauma Center, Hengyang Medical School, Affiliated Nanhua Hospital, University of South China, Hengyang, 421002 China

**Keywords:** COVID-19, SARS-CoV-2, Neuroinvasion, BBB, Cognitive

## Abstract

Coronavirus disease 2019 (COVID-19), caused by severe acute respiratory syndrome coronavirus 2 (SARS-CoV-2), has led to a global pandemic. Although COVID-19 was initially described as a respiratory disease, there is growing evidence that SARS-CoV-2 is able to invade the brains of COVID-19 patients and cause cognitive impairment. It has been reported that SARS-CoV-2 may have invasive effects on a variety of cranial nerves, including the olfactory, trigeminal, optic, and vagus nerves, and may spread to other brain regions via infected nerve endings, retrograde transport, and transsynaptic transmission. In addition, the blood–brain barrier (BBB), composed of neurovascular units (NVUs) lining the brain microvasculature, acts as a physical barrier between nerve cells and circulating cells of the immune system and is able to regulate the transfer of substances between the blood and brain parenchyma. Therefore, the BBB may be an important structure for the direct and indirect interaction of SARS-CoV-2 with the brain via the blood circulation. In this review, we assessed the potential involvement of neuroinvasion under the SARS-CoV-2 infection, and the potential impact of BBB disorder under SARS-CoV-2 infection on cognitive impairment.

## Introduction

At the end of 2019, a large number of cases of pneumonia were reported, and then the disease spread rapidly and was named COVID-19 [1]. As of June 23, 2022, the COVID-19 pandemic has infected nearly 541 million people worldwide and killed 6.32 million people [2]. Since the outbreak of the COVID-19 pandemic, the spread of severe acute respiratory syndrome coronavirus 2 (SARS-CoV-2) has had a dramatic impact on global healthcare systems and socioeconomics [3, 4]. With the application of high-efficiency vaccines, the number of COVID-19 survivors has gradually increased, and people have begun to pay attention to the long-term or delayed sequelae that are caused by SARS-CoV-2 infection, commonly known as “long-COVID” syndrome [5, 6]. Although COVID-19 was initially described as a respiratory disease, data suggest that, depending on the severity of COVID-19 symptoms, 30% to 80% of patients develop neurological complications, and some are sufficiently disabling [7–10]. Therefore, understanding the invasion and impact of SARS-CoV-2 on the central nervous system (CNS) is crucial for future studies.

The vasculature of the CNS is the main channel for blood molecules to enter the brain and tightly regulate the movement of ions, molecules and cells between the blood and the brain, known as the blood–brain barrier (BBB) [11–13]. The BBB is mainly composed of brain endothelial cells, vascular basement membrane, pericytes, astrocyte end-foot, microglia and neurons [11, 14]. These structures act as a bridge between the brain parenchyma and the cerebrovascular system and are collectively referred to as the neurovascular units (NVUs) [15, 16]. BBB endothelial cells are sealed by tight junction (TJ) proteins (ZO scaffolding proteins, claudin-5, and occludin) and junctional adhesion molecules to limit the extracellular and transcellular diffusion of molecules in the CNS [17–19]. Thus, in addition to neuroinvasion, disruption of BBB integrity during COVID-19 may expose the brain parenchyma to SARS-CoV-2 in infected blood, which may affect neuronal activity in the CNS.

Although the cause of neuroinvasion and BBB damage in COVID-19 is still unclear, the extent of neurological injury and BBB damage appears to be related to the degree of cognitive impairment and severity of COVID-19 infection. Cranial nerves and the BBB may be important structures for direct and indirect interactions between SARS-CoV-2 and the brain. In this review, we assessed the potential involvement of neuroinvasion and BBB dysfunction in SARS-CoV-2 infection, exploring its impact on COVID-19-related cognitive dysfunction.

## Clinical evidence of cognitive impairment associated with COVID-19

The lungs are the most severely affected organ in COVID-19, which manifests as diffuse alveolar damage, hyaline membrane formation, inflammatory cell infiltration, and microvascular damage [20]. Although SARS-CoV-2 infection was initially thought to be limited to the respiratory tract, causing severe respiratory syndrome, it was later found that the virus can invade other organs, including the CNS [10, 21]. After analysing data from 214 patients from 67 studies, Motalvan et al. [22] found that 36.4% of patients with COVID-19 developed neurological symptoms (Table 1). Multiple retrospective cohort studies of COVID-19 survivors found that one-third of the patients developed neurological or psychiatric symptoms 6 months after SARS-CoV-2 infection [23, 24]. In addition, multiple studies have shown a high incidence of cognitive impairment in post-COVID-19 patients, exceeding 50% in all studies reporting prevalence [25–30] (Table 1). Notably, cognitive impairment appears to be more common in critically ill patients. In a cohort study of 1438 COVID-19 survivors, Liu et al. found that 10% of severe COVID-19 survivors had dementia and 26.54% had MCI at 6 months after discharge [31]. At 12 months, the number of dementia patients increased to 15%, while the number of MCI patients remained at 26.15%, higher than nonsevere cases (0.76%) and MCI (5.35%) [31] (Table 1). The presence of abnormal brain magnetic resonance imaging (MRI) findings in COVID-19 patients and the detection of SARS-CoV-2 RNA in cerebrospinal fluid may support the possibility that SARS-CoV-2 has neuroinvasive ability [32–35]. Taken together, SARS-CoV-2 enters the brain parenchyma, which may lead to damage and loss of brain neurons and endothelial cells, thereby causing COVID-19-related neurological symptoms.

**Table 1:** Summary of neurological involvement in COVID-19 patients in existing studies

Number of patients	Incidence of cognitive impairment	Other types of neurological symptoms	References
214	–	Headache, disturbance of consciousness, neuralgia, ataxia, acute cerebrovascular disease, seizures	[22]
29	59–65% (at 4 months)	Executive dysfunction (33%)	[25]
2103	61.5–80% (at 3 months)	–	[26]
53	61.5%	Hyposmia (26%), headache (21%), ischaemic stroke (11.1%), coordination deficits (74%), paresis (47%), abnormal reflex status (45%), sensory abnormalities (45%)	[27]
26	69.2%	Anosmia (34%), hyposmia (52%), hypogeusia (100%)	[28]
179	58.7% (at 2 months)	Impaired immediate verbal memory and learning (38%), delayed verbal memory (11.8%), verbal fluency (34.6%) and working memory (executive function) (6.1%)	[29]
226	78%	Impaired executive function (50%), impaired psychomotor coordination (57%)	[30]
1438	Dementia: 10% (at 6 months)–15% (at 12 months) MCI: 26.54% (at 6 months)–26.1% (at 12 months)	–	[31]

The table includes some summaries of neurological involvement in COVID-19 patients in existing studies. The incidence of these symptoms varied with sample size and duration of observation. However, cognitive impairment and other neurological symptoms in COVID-19 patients cannot be ignored

Furthermore, in severe cases of COVID-19, SARS-CoV-2 infection can trigger systemic inflammation and cytokine storms [36]. Significantly elevated levels of interleukin-6 (IL-6) and tumour necrosis factor-α (TNF-α) were found in the cerebrospinal fluid of patients affected by COVID-19 [37–39]. In vivo and in vitro studies have shown that IL-6 and TNF-⍺ can trigger stress response mechanisms that disrupt synaptic plasticity, memory formation, and hippocampal neurogenesis [40–42]. Viruses can cause brain dysfunction and neuronal damage through direct cytolysis or secondary inflammatory responses (indirect effects) [43]. Regardless of whether the brain is affected by SARS-CoV-2 through primary or secondary pathways, the neurological complications of COVID-19 may be related to the invasive effects of SARS-CoV-2 on brain tissue.

## Potential pathways of SARS-CoV-2 invading the CNS

Histopathological studies have shown that SARS-CoV-2 is present in different types of brain parenchyma cells. The underlying neurotropic mechanism of SARS-CoV-2 is not fully understood [44–46]. However, the neurotropic mechanisms previously found in SARS-CoV and other viruses can serve as a reference for evaluating the mechanisms of SARS-CoV-2. According to the current research, there may be two main routes of virus entry into the CNS: neuroinvasive mechanisms and haematogenous transmission routes [47–51].

### SARS-CoV-2 enters the brain along the olfactory nerve

The olfactory nerve is mainly composed of olfactory receptor neurons and directly connects the nasal cavity and the CNS [52]. Some pathogens can infect olfactory sensory neurons and their axons that project into the olfactory bulb, which allows the viruses to bypass the BBB and reach the CNS through the so-called olfactory pathway [53]. In one study, of 38 patients with confirmed COVID-19, 73.7% were reported to have positive RT-PCR tests on nasopharyngeal swabs [54]. In a Spanish COVID-19 cohort, 36% of the patients initially presented with anosmia [55]. In a large European multicentre cohort of mild-to-moderate COVID-19 patients, 85.6% and 88.8%, respectively, had olfactory and taste dysfunction [56].

**Fig. 1:**
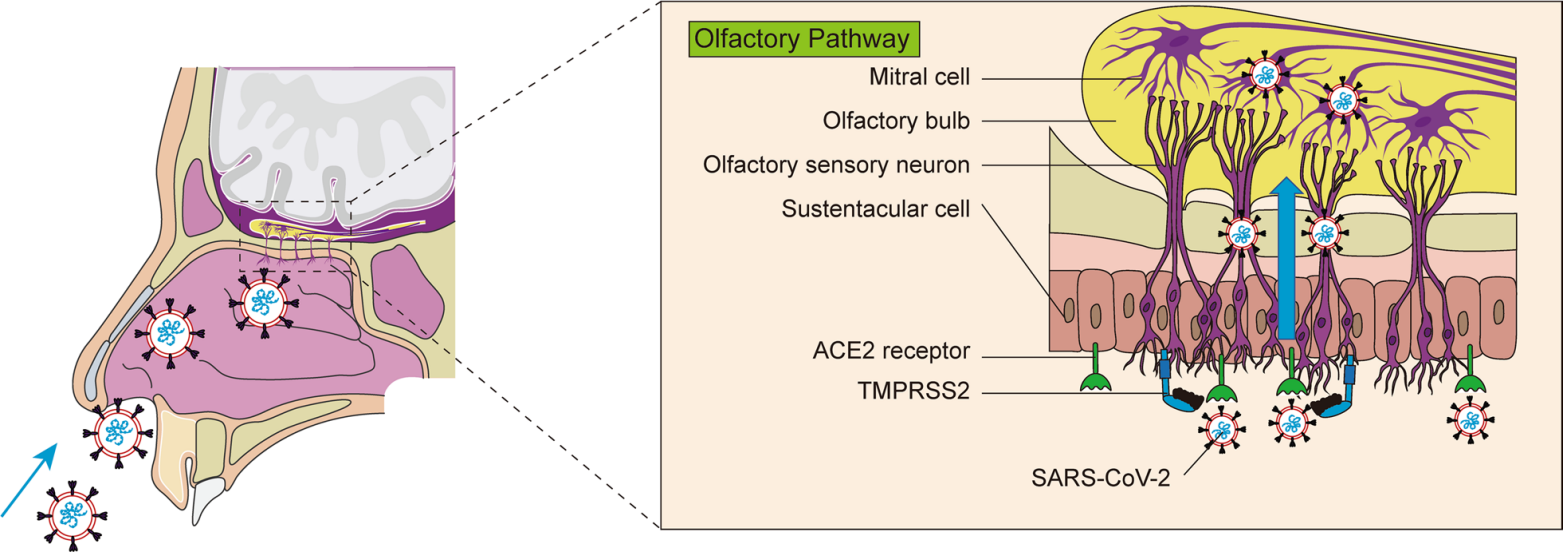
Potential routes of SARS-CoV-2 entry into the brain via the olfactory pathway. Once SARS-CoV-2 is inhaled into the nasal cavity, the virus spreads to the central nervous system along the retrograde axons of the olfactory nerve via the olfactory epithelial receptors ACE2 and TMPRSS2

Each olfactory receptor neuron projects dendrites into the nasal cavity and extends its axons basolaterally through the lamina cribrosa into the olfactory bulb of the brain [52, 57] (Fig. 1). In this pathway, SARS-CoV-2 in the nasal endothelium may attach to motor proteins along sensory and olfactory nerves to travel to the brain [58]. Two days after intranasal administration of SARS-CoV-2 in hamsters, viral antigens were present in the nasal mucosa, bronchial epithelial cells, and areas of lung consolidation, and the virus could infect hamster olfactory sensory neurons [59, 60]. Studies have shown that olfactory epithelial cells express high levels of angiotensin-converting enzyme 2 (ACE2) and transmembrane protease serine 2 (TMPRSS2) [61–63], which could provide a plausible explanation for COVID-19-related anosmia (Fig. 1). Early animal studies have shown that SARS-CoV can enter the brains of ACE2-transgenic mice via the olfactory bulb and cause rapid, transneuronal spread to relevant regions of the brain where infected neurons are dysfunctional [64]. Given the highly similar pathophysiological pathways between SARS-CoV and SARS-CoV-2, this may explain the high incidence of anosmia in COVID-19 patients [60, 61, 65, 66].

**Fig. 2:**
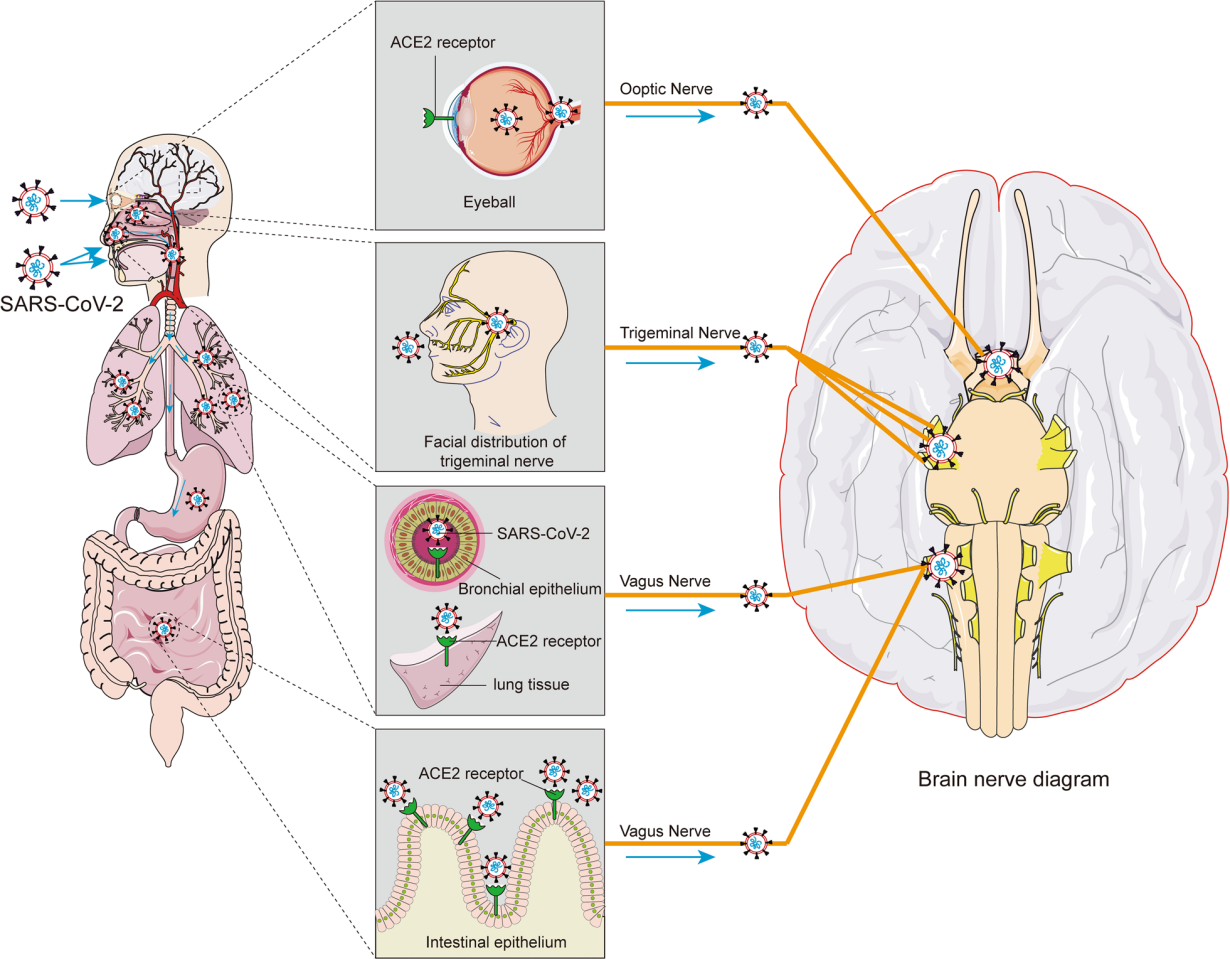
Possible pathways by which SARS-CoV-2 enters the brain through other neural invasions. In addition to infection through the olfactory route, SARS-CoV-2 can also be transmitted through direct or indirect contact with the eyes and oral mucosa. SARS-CoV-2 may enter cells through receptors such as ACE2 on the nasal cavity, eyes, respiratory epithelium, lung parenchyma, and gut and in turn affect multiple cranial nerves (including trigeminal, optic, and vagus nerves). SARS-CoV-2 may infect nerve endings, be transported retrogradely, and spread to other brain regions via synapses

Notably, many research reported that SARS-CoV-2 RNA was detected not only in the olfactory mucosa and olfactory bulb, but also in different branches of the trigeminal nerve [45] (Fig. 2). SARS-CoV-2 may also enter the trigeminal nerve through ACE2 receptor [67]. Afferent fibres from the nasal mucosa travel through the ethmoid nerve to the anterior cranial fossa and travel in the dura to the trigeminal ganglion [68]. Therefore, the strong activation of trigeminal afferents damaged by SARS-CoV-2 may lead to headache and anosmia. The histological changes resulting from intranasal inoculation of neurotropic virus include neuronal and glial necrosis with neutrophil infiltration [69, 70]. Therefore, once SARS-CoV-2 is inhaled into the nasal cavity, the virus may propagate into the CNS along the retrograde axons of the olfactory nerve through the receptors ACE2 and TMPRSS2 on the olfactory mucosa, resulting in neuronal necrosis and dysfunction, thereby causing cognitive impairment due to CNS damage (Fig. 1).

### SARS-CoV-2 transmission through the ocular route

It has been speculated that this may have something to do with the lack of goggle protection [71]. On January 22, 2020, a member of the National Pneumonia Panel was diagnosed with COVID-19 just days after an episode of red eye [71]. The possibility of eye transmission of SARS-CoV-2 is gradually attracting global attention. If a virus can get into our eyes, the most common target may be our conjunctiva [72]. With the development of the epidemic, many COVID-19 patients have developed conjunctivitis as the first symptom or accompanying symptoms [72, 73]. A clinical study of 172 COVID-19 patients showed that the most common ocular symptom in COVID-19 patients was conjunctivitis (23.3%), manifested as conjunctival hyperaemia, foreign body sensation, and itching [74].

However, whether SARS-CoV-2 can be transmitted through the eyes remains controversial [75, 76]. Meinhardt et al. [45] assessed viral load by RT-qPCR on regionally defined tissue samples and found low levels of viral RNA in the corneal, conjunctival and oral mucosa in addition to SARS-CoV-2 in the olfactory mucosa directly below the sieve plate. In addition, immunohistochemical analysis showed that ACE2 was expressed in the conjunctiva, limbus and cornea [77, 78] (Fig. 2). This provides the molecular basis for the susceptibility of the eye to SARS-CoV-2. Recent studies have shown that SARS-CoV-2 can infect the eyes through droplets with viral particles, and the virus can then spread through the nasolacrimal duct to reach the lungs [79]. Inoculation with TCID_50_ of SARS-CoV-2 in the conjunctiva and intratracheal and intragastric inoculation of five rhesus monkeys revealed SARS-CoV-2 [80]. Therefore, the eye has the potential to be a potential infection portal for SARS-CoV-2, supporting the possibility of ocular transmission using the conjunctival mucosa as an entry point for SARS-CoV-2 in the setting of insufficient ocular protection (Fig. 2).

In addition to conjunctivitis, SARS-CoV-2 infection has been associated with lesions leading to visual impairment due to retinal vascular obstruction, ischaemic optic neuropathy, chorioretinitis, and optic nerve inflammation [81, 82]. De Figueiredo et al. [83] described viral arrival at the blood‒retinal barrier, expressing ACE2 and CD147 in retinal pigment epithelial cells and vascular endothelial cells. Because CD147 mediates the disruption of the neurovascular barrier, the virus can cross the bloodstream. CD147 mediates the disruption of the neurovascular barrier, and viruses can cross the blood‒retinal barrier and enter the bloodstream [84]. Therefore, in addition to droplet transmission and direct contact transmission of common respiratory viruses, SARS-CoV-2 may be transmitted to the ocular surface through hand–eye contact and aerosol transmission and then to other systems through the nasolacrimal tract and bloodstream transmission. The potential for ocular transmission of SARS-CoV-2 should not be overlooked. Although haematogenous transfer of SARS-CoV-2 through the eye is theoretically possible, more clinical or experimental evidence is needed to confirm this hypothesis. In the current severe outbreak, more evidence is urgently needed to better assess the potential for ocular transmission and the need for protective measures.

### Neuroinvasion via the vagus nerve

The vagus nerve is the longest nerve in the body and connects vital organs, including the brain, heart, lungs and intestines. In a study of 200 COVID-19 patients, Aranyó et al. [85] found that symptoms such as dizziness, cough, increased heart rate and gastrointestinal problems were associated with damage to the vagus nerve. Although human data are lacking, the vagus nerve complex is known to express ACE2 in rodents [86]. The nucleus solitarius receives sensory information from mechanoreceptors and chemoreceptors in the lung and respiratory tract, so the vagal nucleus solitarius from the lung may be one of the important pathways for virus transport to the brain [87, 88]. Rangon et al. [89] pointed out that SARS-CoV-2 easily invades from the lung along the vagus nerve to the autonomic nerve centre in the brainstem and is involved in the coupling of cardiovascular and respiratory rhythms (Fig. 2). Netland et al. [64] infected brain slices from ACE2 mice with SARS-CoV and found that the dorsal vagal complex, which is critical for cardiorespiratory function, was infected. As reported by Li et al. [90], SARS-CoV-2 migrates from the lungs to the brain and may cause dysfunction of the pulmonary respiratory centre in the brainstem of patients with COVID-19.

In addition, the possibility of enteral infection of SARS-CoV-2 in patients with COVID-19 has also raised concerns [91]. This may be due to the existence of a large number of ACE2 receptors in intestinal epithelial gland cells in addition to the existence of ACE2 in human respiratory epithelium and lung parenchyma [92, 93], which provide the molecular basis for susceptibility to SARS-CoV-2 (Fig. 2).

Therefore, the dorsal vagus nerve complex of the brainstem can be a target of SARS-CoV-2, and the virus may invade brain tissue along the vagus nerve, which may be the neuroanatomical basis for COVID-19 to affect respiration and related reflexes.

## SARS-CoV-2 enters the brain via the BBB

**Fig. 3:**
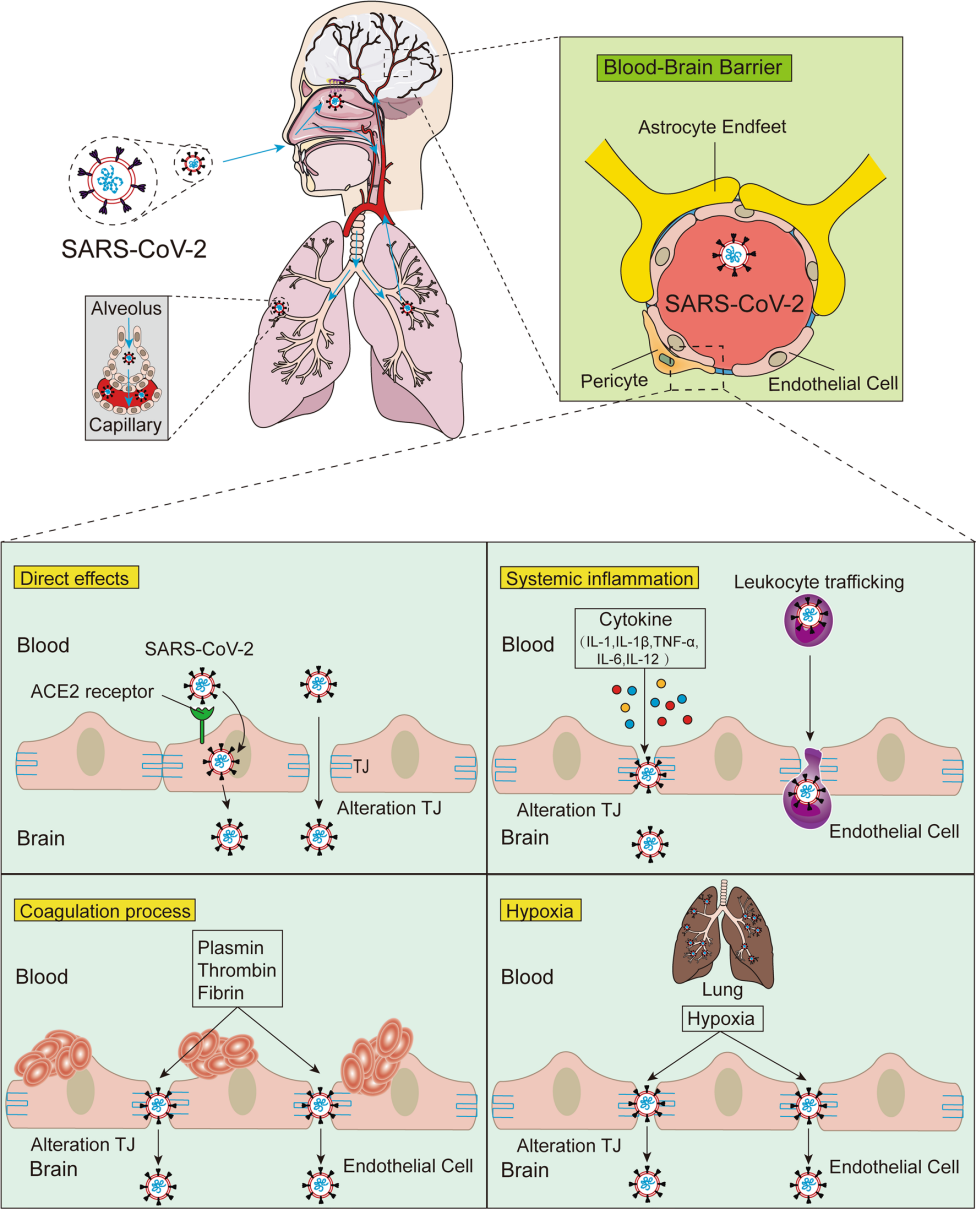
Possible mechanism of SARS-CoV-2 infection of the brain via the haematogenous route. Pulmonary infection with SARS-CoV-2 leads to vascular endothelial damage and increase capillary permeability, allowing virus transfer from the lungs to the pulmonary microcirculation. After reaching the BBB, SARS-CoV-2 may enter the CNS through direct interaction with ACE2 receptors or by altering tight junction proteins formed by BBB endothelial cells. Infection with SARS-CoV-2 increases circulating concentrations of proinflammatory cytokines (IL-1, IL-1β, TNF-α, IL-6, IL-12, etc.), thrombin, fibrinogen, and plasmin, and induced hypoxia to disrupt the BBB may lead to paracellular passage of SARS-CoV-2 as a means of entry into the CNS. In addition, infected leukocytes can carry SARS-CoV-2 across the BBB to infect the CNS through a “Trojan horse” mechanism

In addition to possibly causing brain infection via the neuroinvasive route, SARS-CoV-2 may also enter the brain via the haematogenous route [49, 94]. Studies have pointed out that SARS-CoV-2 is present in the blood of up to 40% of COVID-19 patients [95, 96]. An autopsy analysis of patients who died from COVID-19 showed that SARS-CoV-2 can infect endothelial cells [97]. Normally, viruses such as SARS-CoV-2 cannot easily enter the brain parenchyma through the endothelial cells that surround the capillaries of the systemic circulatory system due to the unique physiology of the BBB. However, the BBB is not impenetrable. BBB disruption and leakage were reported in 58% of COVID-19 patients in 31 case studies of patients with neurological manifestations [98], and these studies provided the first evidence of SARS-CoV-2-induced BBB dysfunction in humans. Disruption of the BBB allows circulating SARS-CoV-2 to invade the brain parenchyma [93, 99, 100]. A recent study in a BBB-on-a-chip in vitro system suggested that the SARS-CoV-2 spike protein may contribute to BBB dysfunction and loss of integrity [101]. The entry of SARS-CoV-2 into the CNS through the BBB may take many forms, some through direct infection and others through secondary mechanisms, such as systemic inflammatory responses and ischaemic and hypoxic changes associated with intravascular coagulation disorders (Fig. 3).

### SARS-CoV-2 directly interacts with components of the BBB

Examination of brain tissue from postmortem SARS-CoV-2-infected patients revealed the presence of viral particles in brain capillaries, endothelial cells, pericytes, and neurons [97, 102, 103]. Studies have shown that SARS-CoV-2 enters cells through the activities of the spike protein, which binds to the ACE2 receptor, and the spike protein is a protein that allows viral RNA to enter healthy cells, allowing the virus to replicate through a complex series of steps [101, 104–106]. The neural transmission of SARS-CoV-2 through the BBB occurs via the infection of vascular endothelial cells due to the presence of ACE2 receptors in endothelial cells [102, 107, 108] (Fig. 3). Once the neurotrophic virus passes through vascular endothelial cells, SARS-CoV-2 invades brain cells through the binding of the S1 subunit of its S protein to ACE2 receptors, which triggers a conformational change in the S2 subunit to achieve membrane fusion with the host cell [109]. The function and overactivity of the ACE2 receptor may affect these target cells and organs, increasing the patient’s susceptibility to infection [110]. The possibility of entry into the cerebrovascular system via other SARS-CoV-2 receptors, such as neuropilin-1 (NRP1) [111] and TMPRSS2 [106], cannot be ruled out. Notably, ACE2 and TMPRSS2 are also expressed in human choroid plexus cells [112]. The choroid plexus has a more permeable blood–cerebrospinal fluid barrier than the tightly regulated BBB and may be a potential site for viral entry into the CNS. Taken together, these data suggest that binding of SARS-CoV-2 with ACE2 receptors on the cerebral vascular endothelium may lead to SARS-CoV-2 crossing the BBB into the brain parenchyma.

Furthermore, viral invasion of the BBB may be associated with disruption of endothelial TJs, leading to BBB dysfunction and enhanced permeability [113, 114]. Using a 3D tissue model of the BBB, the SARS-CoV-2 spike protein was shown to damage endothelial cells and increase the permeability of the BBB [101]. The SARS-CoV-2 spike protein triggers a proinflammatory response in brain endothelial cells, which may lead to alterations in the BBB functional status. Subsequent studies found that the SARS-CoV-2 spike protein led to disruption of the BBB by downregulating TJ proteins in human brain microvascular endothelial cells, resulting in viral entry into the CNS via a paracellular pathway [115, 116] (Fig. 3).

### SARS-CoV-2 infection triggers systemic inflammation and promotes BBB leakage

In severe cases of COVID-19, SARS-CoV-2 infection can trigger systemic inflammation and cytokine storms [36]. Krasemann et al. [117] used an in vitro BCEC model to show intrinsic inflammatory signatures following exposure to SARS-CoV-2. Indirect effects of a hyperinflammatory state may be the mechanism involved in the BBB disruption associated with COVID-19 [118]. Elevated levels of proinflammatory factors are closely related to changes in TJ function and BBB disruption. For example, elevated levels of IL-1 can lead to impaired BBB integrity [119]. IL-1β also activates extracellular signal-regulated kinases, upregulates matrix metalloproteinase (MMP)-9 and disrupts TJ proteins [120, 121]. In addition, cytokines such as TNF-α, IL-6, and IL-12 can lead to the degradation of TJ proteins (occludin, claudin-5, ZO proteins), resulting in impaired BBB permeability [122, 123]. Concomitant with inflammatory damage to the BBB, the extravasation of immune cells through the BBB increases, resulting in increased SARS-CoV-2 viral particles, as well as proinflammatory cytokines, in the brain parenchyma. Exposure of astrocytes to viral particles and proinflammatory mediators triggers the activation of cytokines and the production of vascular endothelial growth factor (VEGF) in these cells [124, 125]. VEGF in brain capillary endothelial cells breaks down TJ proteins by activating the phosphoinositide 3 (PI3)-kinase and AKT signalling pathways and by upregulating MMP-9 protein levels, resulting in BBB leakage [126–128]. Increased secretion of proinflammatory cytokines associated with COVID-19 impairs BBB integrity and accelerates SARS-CoV-2 entry into the brain parenchyma [99, 122, 129] (Fig. 3).

In addition, virus-infected leukocytes spread into the blood circulation and extravasate into the brain parenchyma with other immune cells, and this may be another route for the virus to enter the CNS. Infected leukocytes with neurotropic viruses, such as human immunodeficiency virus (HIV) and West Nile virus (WNV), can infiltrate the brain through the vasculature, meninges, and choroid plexus, and this mechanism is known as the “Trojan horse” [130, 131]. SARS-CoV-2 likely also uses this mechanism to invade the CNS by infecting ACE2-expressing leukocytes. This evidence, combined with the systemic inflammatory and hypoxic conditions in COVID-19, shows that there is increased leukocyte infiltration through the BBB during infection [118, 132], which reinforces this pathway for SARS-CoV-2 to invade nerves (Fig. 3). Experience from cohort observations suggests that persistent systemic inflammation during COVID-19 infection is associated with subsequent cognitive decline [133] and leads to persistent electroencephalography (EEG) changes and hippocampal atrophy [134].

Therefore, in cognitive impairment related to COVID-19, attention should be given to the impact of proinflammatory factors on the body. SARS-CoV-2 infection causes proinflammatory cytokines to activate specific signalling cascades and increase BBB leakage by impairing TJ proteins assembly and expression levels, which in turn allows SARS-CoV-2, peripheral immune cells and other molecules to enter the CNS, thereby exacerbating brain damage.

### SARS-CoV-2 infection leads to coagulopathy, promotes TJ disruption and increases BBB permeability

Acute viral infections, including SARS-CoV-2, have been reported to increase the risk of ischaemic stroke [135, 136]. The severity of COVID-19 is positively correlated with the risk of stroke [51]. Coagulation is frequently impaired in COVID-19 patients, resulting in a common hypercoagulable state in patients, and may be related to the incidence of stroke [137–139]. In two COVID-19 cohorts, the incidence of ischaemic stroke secondary to thromboembolic complications was 1.6% [140] and 2.5% [141]. Critically ill patients infected with SARS-CoV-2 often exhibit elevated d-dimer levels and severe thrombocytopenia, which may increase the probability of cerebrovascular events [95, 142]. Han et al. [143] conducted a prospective retrospective analysis of coagulation data from 94 patients with confirmed COVID-19 and found that d-dimer, fibrin/fibrinogen degradation products and fibrinogen levels were significantly higher in all SARS-CoV-2-infected cases than in the healthy control group.

Using an adult rat model of intraventricular haemorrhage, Liu et al. [144] found that thrombin can disrupt the BBB through a molecule that activates the phosphorylation of Src kinase by its protease-activated receptor. Src family members can increase BBB permeability by phosphorylating MMPs and TJ proteins [145, 146] and upregulating VEGF [147]. In addition, it was shown in vascular endothelial experiments that fibrinogen can damage endothelial cell integrity by disrupting TJ proteins bound to actin filaments [148]. Increased actin formation may lead to cellular stiffness, retraction of actin filaments, and widening of interendothelial junctions, thereby disrupting endothelial cell integrity [149, 150]. By the intraventricular injection of endogenous tissue plasminogen activator (tPA), Yepes et al. [151] found a rapid dose-dependent increase in vascular permeability. Consistent with the features of thrombotic microangiopathy, coagulation factors were elevated in COVID-19 patients with abnormal mental status [138]. Computed tomography (CT) images of a patient with COVID-19 and necrotizing haemorrhagic encephalitis showed symmetrical hypodensity in the bilateral medial thalamus, and MRI confirmed haemorrhagic lesions in the bilateral thalamus, subinsula, and medial temporal lobes [152]. These studies suggest that ischaemia-induced increases in endothelial permeability involve a cascade of events in which the thrombin, fibrinogen and plasmin systems are major players.

In summary, abnormalities in the coagulation system caused by SARS-CoV-2 infection may increase the permeability of the BBB and increase the entry of SARS-CoV-2 into the brain parenchyma by disrupting TJ proteins (Fig. 3). Invasion of vascular endothelial cells by SARS-CoV-2 activates a thrombotic and inflammatory cascade leading to capillary occlusion. For example, brain structures that manage cognition, such as the hippocampus, temporal lobe, and thalamus, are involved, resulting in ischaemia and hypoxia damage to nerve cells nourished by these capillaries, which can promote the occurrence of vascular cognitive impairment.

### Lung injury caused by SARS-CoV-2 leads to hypoxia increasing BBB permeability

Patients with COVID-19 frequently experience severe hypoxia due to respiratory distress, putting them at risk for hypoxia-related encephalopathy [153, 154]. An autopsy analysis of the brains of COVID-19 subjects revealed a very high incidence of acute hypoxic injury [155]. Respiratory failure from lung damage can lead to severe hypoxia in the brain [156]. Neurons rely on blood vessels to provide oxygen and nutrients. When brain tissue is continuously hypoxic, it will eventually lead to irreversible damage to neurons [157]. Consistent with hypoxic brain injury, postmortem studies of COVID-19 have demonstrated neuronal damage in regions of the neocortex, hippocampus, and cerebellum [155, 158, 159]. Autopsy studies have shown that hypoxia can lead to oligodendrocyte death and extensive gliosis [160]. Numerous in vitro and in vivo studies have shown that hypoxia leads to BBB disruption, which may be a trigger for subsequent CNS disease [161].

Chen et al. [132] established an in vitro BBB model by coculturing mouse brain microvascular endothelial cells and astrocytes and found that hypoxia reduces ZO protein expression and induces ZO protein phosphorylation. Furthermore, using primary bovine brain microvascular endothelial cells, Mark et al. [162] found that hypoxia resulted in a 2.6-fold increase in actin rearrangement and the paracellular permeability marker [^14^C] sucrose. These findings are consistent with previous reports showing increased permeability of brain capillary endothelial cells after 2 h to 48 h of hypoxia treatment [163, 164]. Notably, the expression of occludin and ZO proteins increased, while the protein expression or localization of claudin-1 was almost unchanged after hypoxia or reoxygenation [162]. This suggests that claudin may not be involved in the hypoxia-induced changes in paracellular permeability. The hypoxia-induced increase in paracellular permeability of brain capillary endothelial cells may be associated with altered actin distribution and the loss of TJ proteins (Fig. 3). Conditions such as hypoxia, encephalitis, and stroke are known to produce long-term or even permanent neurocognitive impairment [165]. Therefore, some patients with COVID-19 are expected to develop long-term neurocognitive sequelae after the acute disease has resolved. In general, the chronic cognitive sequelae of ischaemia and hypoxia can range from mild attention and memory impairment to general cognitive decline and dementia and even coma.

## Conclusions

Growing evidence suggested that survivors of COVID-19 suffer from neurological involvement. The brain is undoubtedly one of the targets of COVID-19. The exact pathophysiology of CNS infection is currently still speculative but appears to be related to a range of processes, including neuroinvasion and the effects of systemic infection consequences, both of which trigger BBB dysfunction, neuroinflammation, ischaemia and hypoxia and thus lead to secondary infections and brain dysfunction. The infection mechanism of COVID-19 in the brain may be related to the high-density expression of ACE2 receptors in the brain and other organ tissues and the entry of the virus into the brain through the olfactory nerve, trigeminal nerve, optic nerve and vague nerve pathways. Another blood-borne route is also possible, which involves viruses crossing the BBB. Mechanisms by which SARS-CoV-2 interacts with the BBB may lead to the neurological dysfunction that is associated with SARS-CoV-2-induced COVID-19. Recent information suggests that SARS-CoV-2 is able to infect CNS cells, especially the brain microvascular endothelial cells of the BBB. The effects of SARS-CoV-2 on the CNS may cause acute and long-term changes in the nervous system or could exacerbate existing neurological diseases or symptoms. Therefore, neuroinvasion and BBB dysfunction may be potential pathways that promote SARS-CoV-2 entry into the CNS and may contribute to the cognitive impairment observed during disease progression.

Notably, it is important for SARS-CoV-2 models to reliably test the molecular and functional consequences of infection and drug treatment strategies via the establishment of a high paracellular tightness in vitro that is comparable to physiological conditions in vivo. Currently, the link between BBB leakage and cognitive decline is poorly understood, and more research is needed to further define this link. Furthermore, COVID-19 will continue to affect the health of the body long after the epidemic ends, so continuous assessment of an individual's susceptibility to cognitive decline and dementia in the future will be important to improve patients’ quality of life later in life. Although it is too early to elucidate the long-term side effects of SARS-CoV-2 infection, growing evidence pointed that SARS-CoV-2 may lead to permanent sequelae of the CNS, including cognitive decline. However, whether SARS-CoV-2 could enter the brain and replicate in the brain parenchyma, whether it has neuroinvasive capabilities, should be explored in future. In summary, with the advent of the post-epidemic era, the subsequent brain damage caused by SARS-CoV-2 will become a clinical symptom and social problem that cannot be ignored. The exploration of the mechanism on cognitive impairment in patients with COVID-19 and the early intervention will improve patient's life quality in future.

## Data Availability

This review does not contain any analysable data. All authors cited in this paper are publicly available.

## Abbreviations

COVID-19: Coronavirus disease 2019
SARS-CoV-2: Severe acute respiratory syndrome coronavirus 2
BBB: Blood–brain barrier
NVUs: Neurovascular units
CNS: Central nervous system
TJ: Tight junction
JAMs: Junctional adhesion molecules
MRI: Magnetic resonance imaging
Aβ: Amyloid-beta
IL-6: Interleukin-6
TNF-α: Tumour necrosis factor-α
ACE2: Angiotensin-converting enzyme 2
TMPRSS2: Transmembrane protease serine 2
NRP1: Neuropilin-1
MMP: Matrix metalloproteinase
VEGF: Vascular endothelial growth factor
NO: Nitric oxide
PI3: Phosphoinositide 3
HIV: Human immunodeficiency virus
WNV: West Nile virus
EEG: Electroencephalography
tPA: Tissue plasminogen activator
CT: Computed tomography
